# Ambulatory second look percutaneous nephrolithotripsy with maturated nephrostomy tract

**DOI:** 10.1590/S1677-5538.IBJU.2019.0003

**Published:** 2020-08-10

**Authors:** Hyun Suk Yoon, Wan Song, Kwang Hyun Kim, Hana Yoon, Dong Hyeon Lee, Woo Sik Chung, Bong Suk Shim, Jeong Hwan Son

**Affiliations:** 1 Department of Urology Ewha Woman’s University School of Medicine Republic of Korea Department of Urology, Ewha Woman’s University School of Medicine, Republic of Korea;; 2 Department of Urology Bundang Jesaeng Hospital Republic of Korea Department of Urology, Bundang Jesaeng Hospital, Republic of Korea

## Abstract

**Introduction and Objectives:**

Percutaneous nephrolithotomy (PCNL) is the standard technique for managing large renal calculi. Second-look PCNL is typically performed under intravenous (IV) sedation or spinal / general anesthesia when removing remnant stones. This requires additional pre-anesthesia assessment and close monitoring. To simplify this procedure, we investigated the feasibility and safety of second-look PCNL without anesthesia and sheath after maturation of the nephrostomy tract.

**Material and Methods:**

This study included 14 eligible patients with remnant stones >5mm in diameter, as determined by simple CT scan after supine PCNL through a single nephrostomy tract under general anesthesia. A 24Fr nephrostomy tube was inserted after surgery. Second-look PCNL was performed after seven days of maturation of the nephrostomy tract. Prior to second-look surgery, 25mg pethidine was injected intravenously. Second-look supine PCNL was performed using a rigid or flexible renoscope without anesthesia or sheath.

**Results:**

The mean patient age was 57.4±8.5 years. The mean stone diameter was 5.4 × 3.3cm, while the mean number of stone branches was 4.1±1.4. The mean operation time during the first PCNL was 131.1±24.8 min, and the mean residual stone rate was 24.3%±10.2%. The mean operation time during second-look PCNL was 97.4±36.0 min; after the second procedure, the mean pain score on the numeric rating scale was 2.8±1.0. All patients were stone-free without complications.

**Conclusion:**

Second-look PCNL without anesthesia and sheath after maturation of the nephrostomy tract may be an effective procedure for removing remnant stones in select patients without excessive levels of pain.

